# Return Cases of Scarlet Fever

**Published:** 1901-03-09

**Authors:** 


### RETURN CASES OF SCARLET FEVER.

Without quoting the whole of the conclusions arrived at by Professor Simpson as the result of his inquiry into the return cases of scarlet fever and diphtheria traceable to cases discharged from the hospitals of the Metropolitan Asylums Board, we may indicate some of the more important of them.

It is shown that 80 per cent. of the primary infective cases are connected with discharges from mucous membranes, the discharges from the nose in scarlet fever forming 40 per cent. of the cases, and in diphtheria 28 per cent.

The administration of warm baths immediately before the patient is sent out of hospital is condemned, on the one hand because it does not remove the infection, and on the other because, combined with the exposure in the winter to the vicissitudes of the weather, it contributes to increase of the infection, and brings back rhinorrhœa and otorrhœa. This, as it seems to us, is a very important matter. Such bathing just before going out would never be done in private practice, and although no doubt it has been done in the hope of affording protection to the public, it must be wrong.

It is held that mere duration of detention in hospital is no standard as to the limit of infection, and that long detention in infective wards will not reduce the percentage of return cases. This, again, is a matter of very great importance, for no one can doubt that for a long time past the resources of the hospitals have been much crippled by the length of time during which it has been customary to detain scarlet-fever patients in the hope of their infectivity passing away.

Clinically, a matter of much interest is the statement that the discharges from mucous membranes on which the infection has been sown are probably the carriers and not the causal agents of infection, and that local and antiseptic treatment, combined with transference to less infective wards, are likely to be more effective than long detention in reducing the percentage of return cases. Many people have regarded the discharge itself as the infective agent, as if the discharge were an indication of the continuance of a "scarlatinal catarrh" or a "diphtheric catarrh," a constant shedding of infective matter. Dr. Simpson, however, has come to the conclusion that the infectivity of a chronic mucous discharge is not due to the secretion itself, but to the mico-organism of the disease, being accidentally grafted on to the secretion owing to the patient continuing to live in an infected place. As he says, (*a*) the infection does not continue long when the patient returns home though the discharge continues; (*b*) when there is a recurrence of the discharge some time after the return home of the patient from hospital the patient is not infective; and (*c*) a large number of children with otorrhœas and with discharges from the nose go home without infecting anybody else.

It is shown that the time during which patients are retained in hospital (which sometimes extends to twelve or fifteen weeks) far exceeds the time during which patients treated at home are quarantined, and it is held that while the isolation at home is insufficient in duration, that in hospital is, if anything, too long; and, further, that return cases are not due to premature discharge from hospital.

A special committee of the Royal College of Physicians has also considered this question of return cases, and has recommended that a couple of experiments should be carefully tried: 1. That a couple of wards in each hospital identical in respect to cubic space, &c., per bed, and the age and sex of the children, should be set aside and administered on a different principle; that from one ward all cases subject to mucous discharges from nose or ear should be rigorously excluded; that immediately on the appearance of any such discharge the patient should be removed; and that all nozzles of syringes should be kept in antiseptic solutions. In the other ward no special attention should be paid to these discharges other than that hitherto adopted. The incidence of either rhinorrhœa or otorrhœa should then be compared. Cases of "septic scarlet fever" should be excluded from both alike; 2, that in certain hospitals two or more rooms, previously disinfected, be reserved for the isolation, after six or eight weeks of detention, of single patients who are suffering from rhinorrhœa or otorrhœa, but whose desquamation is completed. Each patient so secluded should be kept for ten days or a fortnight before returning home. During this detention the affected parts should be regularly irrigated or syringed with some reliable germicidal solution, and at the end of the time the patient should be sent home, whether the discharge has ceased or not. The room should be disinfected before being used by another patient, and the subsequent history of each case should be investigated.

The Metropolitan Asylums Board have given authority to medical superintendents at their several hospitals to carry out these experiments.

